# Factors associated with family planning use among refugee and host populations in Adjumani district, West Nile, Uganda: a comparative study

**DOI:** 10.1186/s12889-024-18103-x

**Published:** 2024-03-11

**Authors:** Roselline Achola, Lynn Atuyambe, Elizabeth Nabiwemba, Makumbi Fredrick, Christopher Garimoi Orach

**Affiliations:** 1 Department of Community Health and Behavioural Sciences, School of Public Health, College of Health Sciences, Makerere University, P.O Box 7072, Kampala, Uganda; 2 Department of Epidemiology and Biostatistics, School of Public Health, College of Health Sciences, Makerere University, P.O Box 7072, Kampala, Uganda

**Keywords:** Decision making, Family planning use, Factors associated, Refugees, Host, Uganda

## Abstract

**Introduction:**

Uganda currently hosts an estimated 1.5 million refugees. The refugees have challenges in accessing family planning (FP) services in the host country. The study aimed to investigate factors associated with FP use among host and refugee populations in Adjumani district, Uganda.

**Methods:**

A comparative cross-sectional study was conducted in May 2021 in three refugee settlements and their host communities in Adjumani district. A total of 1,310 respondents, (664 refugees and 646 host) were randomly selected using multistage cluster sampling and interviewed. Quantitative data were collected using structured questionnaires and analyzed using STATA V.15. Descriptive and Multivariate analysis performed.

**Results:**

We found that modern Contraceptive Prevalence Rate (mCPR) was 30.2% (32.2% for host and 28.2% for refugees). Multivariate analysis showed that women who live singly (AOR = 2.25, 95%, CI: 1.56 -3.84), completed primary education [AOR = 1.65, 95% CI: 1.27–2.16], acquired skills [AOR = 2.28, 95% CI: 2.11–2.47], have the desire for another child [AOR = 3.73, 95% CI: 1.45- 9.60], have stayed in the study area between 3–5 years [AOR = 2.24, 95% CI: 1.46–3.42] were statistically significantly associated with FP use among both refugee and host populations. The key barrier to FP use by host was harassment of women and separation/divorce for not consulting the family members. Whereas amongst the refugees, they do not want to use FP methods.

**Conclusion:**

Our findings revealed low FP use amongst both populations in Adjumani district. The main factors associated with FP use amongst refugee populations included marital status, level of education, type of occupation, and duration of stay in the study area whereas amongst the host is the marital status. Main reasons for not using FP methods included fear of side effects by hosts and not wanting to use FP by refugees. There is need to sensitize both communities about the benefits of FP at community level.

## Introduction

Globally, there are an estimated 89.3 million people displaced [1]. Over half of the world’s refugee population come from Syria, Afghanistan and South Sudan [2]. Uganda currently hosts an estimated 1.5 million refugees and is the leading refugee-hosting country in Africa and 3rd largest refugee hosting nation in the world [3, 4]. In Uganda, 94% of the refugees live in settlements established in 12 districts of Adjumani, Arua, Yumbe, Obongi, Koboko, Madi Okollo, (West Nile region); Lamwo, Kiryadongo (Northern region), Kyeggegwa, Isingiro, Kikube, Kamwenge (Western Region). The majority of the refugees in Uganda come from South Sudan (60.5%, *n* = 964,960). Others come from Democratic Republic of Congo (29.3%, *n* = 467,004), and the rest come from Somalia, Burundi, Rwanda, Eritrea, Ethiopia and Sudan (10.2%, *n* = 163,441) [1].

Family Planning [FP] is a fundamental human right and is central to reproduction [5]. It may be one of the most life-saving, empowering and cost-effective interventions for women and girls [6]. However, it remains underfunded with limited prioritization in humanitarian responses [7]. Although expenditure on Global Humanitarian emergencies has significantly increased from US$18 billion in 2005 to over US$ 28 billion to date [8], limited budget is earmarked for FP services. Additionally, FP services are limited due to the fragile nature of the settings for both refugees and service providers [9]. Whereas it is important to increase the use of FP services among populations in the humanitarian settings, their ability to use any method is affected by several barriers [10]. These barriers include language, low educational level, lack of information, influence by significant others, limited income, desire to replace lost family members, moral values, certain taboos, cultural norms, religious impediments and personal experience with contraceptives side effects [11]. These barriers have contributed to high unmet need for FP among women and girls leading to high rates of unintended pregnancies. In addition socio-cultural preference and unacceptability of contraception also pose significant barriers to making decision to use FP [6, 7]. Other structural barriers that affect use of FP by refugees in humanitarian settings include lack of privacy, weak supply chain system leading to unavailability of a range of methods of FP and regular stock outs, weak service provision with poor quality of care for women and girls leading to long waiting hours [12].

Amongst the host population, studies by [13, 14] have reported several factors that affect FP use. These include weak health systems, poor services, myths and misconceptions, lack of partner support and socio-economic factors. However, in developing countries, over 200 million women who wish to avoid next pregnancy lack access to their preferred choice of FP method [15]. Therefore, providing women with choice is important in promoting the use of family planning and consequently reducing the unmet need [16]. In Uganda the modern Contraceptive Prevalence Rate (mCPR) stands at 38% with high-unmet need for FP at 22% [17]. While the mCPR in Adjumani district is 25%, the adolescent birth rate is 20% with Maternal Mortality Ratio of 600/100,000 live births according to Adjumani district development plan of 2020 which is far beyond the national MMR of 189/100,000 live births [18]. The total fertility rate in Adjumani is estimated at 8.9% with an annual growth rate of 6.4%, which is over two-fold the national annual growth rate of 3.0% [17].

Several studies have shown the possibility, feasibility, and strategies of providing FP services in humanitarian settings [19]. However, there are other factors that continue to affect use of contraception in humanitarian settings such as socio-cultural norms, low partner support among others. Therefore, FP methods should be culturally and socially acceptable by all the ethnic and religious groups within the refugee settlements and the existing structures be strengthened and used to expand access and availability of FP services. While some scholars have studied systems decision making which includes decision making units and decision maker [20, 21], it is however, not clearly known how individuals decide to use FP in a humanitarian settings. Therefore, this study established factors associated with family planning use amongst refugee and host populations in Adjumani district.

While contraception is critical in saving the life of a mother by preventing unplanned pregnancies and unsafe abortion as well reducing the risk of children dying, providing choice for women to make decision for their FP method may lead to various achievements [22].

The achievements include; family stability, getting better attention with partners and better child mother bonding as a result of spacing [23].

## Methods

### Study setting

The study was conducted in Adjumani refugee affected district in west Nile region, Uganda. Adjumani district is located on the eastern bank of the Albert Nile [ADDP, 2020]. The district has a total land area of 3128 square kilometers, with 46.8 square kilometers covered by water, 37.44 square kilometers is occupied by forest and 1455 square kilometers is arable land [ADDP, 2020]. The ethnic background of the people in Adjumani are Madi, Acholi, Lugbara and Kuku who live both in the banks of River Nile in Uganda and South Sudan [24]. The refugees from South Sudan have been hosted in the West Nile region with majority in Adjumani district since 2013 due to their similarities with the local population and the propinquity. They have diverse ethnic backgrounds including the Dinkas, Kuku, Nuer, Kakwa, Madi, Siluk, Acholi among others. They speak the central Sudanic language of the Nilo Saharan language family. Adjumani district hosts more refugees 244,374 (50.7%) than the indigenous population of 237,400 (49.3%) inhabitants. Among the refugees, 85% (182,977) are women and children. This study was conducted in three settlements namely, Nyumanzi, Pagirinya and Mirieyi including the surrounding host communities. These settlements were purposively selected. The two settlements (Nyumanzi, 40,877 and Pagirinya, 36,784) have the highest number of refugees in the district and have existed for more than 23 years. The third and newest settlement established in Adjumani is Mirieyi with over 7,067 refugees.

### Study design

This was a comparative cross- sectional study. The study population were women of reproductive age (15—49 years) and men of ages 18—65 years.

### Sample size determination

The number of participants per group required to detect a difference proportions (P_1_ and P_2_) with a 5% level of significance (α) and 80% power (1-β) was estimated using the formula below;

$$\frac{n={({{\text{P}}}_{1}(1-{{\text{P}}}_{1}) + {{\text{P}}}_{2}(1-{{\text{P}}}_{2})) ({{\text{Z}}}_{1-\mathrm{ \alpha }/2}+ {\mathrm{Z }\,1}_{-\upbeta })}^{2}}{{({{\text{P}}}_{1}-{{\text{P}}}_{2})}^{2}}$$

Where P_1_ is the proportion of FP use in host population (25%) and P_2_ is the expected proportion of FP use in refugee populations (15%). This gave a sample size of 456 per group and 992 overall. However, the multistage cluster sampling employed for respondent’s selection resulted into the sample size of 1,310. Therefore, a total of 1,310 respondents were sampled and interviewed. The host comprised of 646 and the refugees were 664.

### Sampling procedure

Multistage cluster sampling technique was used to select both refugee and host populations.

### Sampling of refugee populations

Out of the nineteen settlements, three were purposively selected given the longevity of establishment (Pagirinya and Nyumazi) and the latest establishment (Mirieyi) for better representation. Simple random sampling was used to select the three clusters/zones, villages, blocks and households from the selected settlements. Simple random sampling was used to select participants from the list of households identified. Clustering was done in various stages. In stage 1, the settlements were clustered in zones and one zone was selected randomly, giving a total of 3 zones. In stage 2, two villages were selected randomly from the 3 zones, giving a total of 6 villages. In stage 3, three blocks were selected randomly from the 6 villages, giving a total of 18 blocks. In stage 4, thirty-five households per block were randomly selected from the 18 blocks giving a minimum Sample size of 630 households for the study. However, 646 respondents were interviewed.

### Sampling of host populations

For the host population, two counties were purposively selected. Simple random sampling was used to select sub counties, parishes, villages and households. Adjumani district and the two counties were selected purposively. The sampling was done in stages as below:

In stage 1, two sub counties were randomly selected from 2 counties, giving a total of 4 sub counties. In stage 2, two parishes were randomly selected from the 4 selected sub counties, giving a total of 8 parishes. In stage 3, two villages were randomly selected from the 8 selected parishes, giving a total of 16 villages. In stage 4, forty-one households were randomly selected from the 16 selected villages, giving a minimum sample of 656 households. However, we interviewed 664 respondents from the host population. Overall, 1,310 respondents were interviewed.

For both host and refugee communities, RAs first obtained the list of all the blocks/villages and households. The households were randomly selected from which women and men were interviewed. For every 2 women interviewed, the 3rd was an eligible man. A household-to-household surveys was conducted until the required number of respondents (women and men) were achieved from both populations. This was applied at the village level to select households. We considered the number of households per village/blocks that were proportionate to size.

### Data collection procedures

Data were collected through surveys from both refugee and host populations (men and women) using translated pre-tested structured questionnaires. The research assistants were trained for 4 days prior to data collection. From the selected households, one woman was interviewed. We interviewed respondents in a secure place of their choice to guarantee privacy and confidentiality. Interviews were conducted in local languages of Arabic and Madi that were best understood by the refugees and host population respectively. Data were collected using the Open Data Kit (ODK).

The research protocol and data collection were approved by Makerere University School of Public Health Higher Degrees Research and Ethics Committee (HEDREC) #188 and the Uganda National Council of Science and Technology.

### Data management and analysis

Data were imported into and analyzed using STATA software Version 15. The data were reviewed for completeness, consistency, and accuracy. This study focuses on the outcome variable of family planning use. Current FP use was defined as any respondent currently using any method of FP. The independent variables included in the regression models were factors assumed to influence FP use such as sex, age, peers, ethnic group, refugee status, socio-economic status, religion, education, marital status, duration of stay as refugee and number of living children. Descriptive analysis and Multivariable Logistic regression were performed to establish factors associated with FP use among refugee and host populations. Statistical significance was set at 5%. Multilevel sampling and clustering were accounted for by obtaining robust standard errors. Multicollinearity was ruled out using Variance inflation factors (VIF), all the independent variables had VIF less than 5.

## Results

The total number of respondents interviewed from refugees were 664 and from hosts were 646 (Table 1). Majority of respondents were females [63.7%, *n* = 835]. More than half of the respondents [38.9%, *n* = 509] were in the age group of 25–34 years. The mean age for respondents in the host communities was 32.34 years, SD 10.30. While the mean age for the refugees was 30.67 years, SD 10.07. Two thirds [67.3%, *n* = 881] of the respondents were in monogamy marriage/cohabiting. A higher proportion of respondents [41.8%, *n* = 547] had no formal education or did not complete primary. A higher proportion of the refugee [40.7%, *n* = 270] compared to host respondents [30%, *n* = 194] had attained Secondary education and beyond. More than half of the respondents [66%, *n* = 721] were Catholics compared to [25.6%, *n* = 335] who were Anglicans.

**Table 1:** Socio-demographic characteristics of respondents

Variables	Refugee		Host		Total	
	**n**	**%**	**n**	**%**	**N**	**%**
**No of respondents**	**664**	**100**	**646**	**100**	**1,310**	**100**
**Sex**						
Male	217	32.7	258	39.9	475	36.3
Female	447	67.3	388	60.1	835	63.7
**Age group**						
15–24 years	201	30.3	159	24.6	360	27.5
25–34 years	259	39.0	250	38.7	509	38.9
35–45 years	130	19.6	154	23.8	284	21.7
45-54 years	56	8.4	57	8.8	113	8.6
55 & more years	18	2.7	26	4.0	44	3.4
**Marital status**						
Never married	119	17.9	42	6.5	161	12.3
Married/Monogamy	411	61.9	470	72.8	881	67.3
Married/Polygamy	101	15.2	95	14.7	196	15.0
Widowed/Divorced/Separated	33	5.0	39	6.0	72	5.5
**Education**						
None/Incomplete Primary	257	38.7	290	44.9	547	41.8
Completed Primary	137	20.6	162	25.1	299	22.8
Secondary and beyond	270	40.7	194	30.0	464	35.4
**Religion**						
Catholic	249	37.5	472	73.1	721	55.0
Anglican	226	34.0	109	16.9	335	25.6
Pentecostal	54	8.1	32	5.0	86	6.6
Muslim	38	5.7	13	2.0	51	3.9
SDA	14	2.1	20	3.1	34	2.6
ECS	83	12.5	0	0.0	83	6.3
**Occupation**						
Peasant	506	76.2	473	73.2	979	74.7
Semi-skilled	130	19.6	136	21.1	266	20.3
Skilled	28	4.2	37	5.7	65	5.0
**Duration of stay in area**						
< 3 years	73	11.0	66	10.2	139	10.6
3–5 years	325	48.9	77	11.9	402	30.7
6 & more years	265	39.9	489	75.7	754	57.6
**Want another child**						
No	127	19.1	158	24.5	285	21.8
Yes	537	80.9	488	75.5	1,025	78.2
**Sources of FP information**						
Health worker	490	73.8	519	80.3	1,009	77.0
Peers	31	4.7	10	1.5	41	3.1
VHTs	52	7.8	50	7.7	102	7.8
Other sources	41	6.2	5	0.8	46	3.5
**Sources of FP services**						
Hospital	570	85.8	599	92.7	1,169	89.2
Private Clinics	36	5.4	3	0.5	39	3.0
Drug-Shops	5	0.8	4	0.6	9	0.7
VHTS	31	4.7	37	5.7	68	5.2
Other sources	22	3.3	3	0.5	25	1.9

We also established that the occupation of the respondents was mainly peasant [74.7%, *n* = 979] with majority [76.2%, *n* = 506] from the refugee population (Table 1). The study also revealed that over 48% of refugees had lived in the settlement in Adjumani district between 3–5 years. Overall, our study established that [78.2%, *n* = 1025] wanted to have another child. Majority of respondents who wanted to have another child were amongst refugees [80.9%, *n* = 537] as compared to those from the host populations [75.5, *n* = 488]. Although various sources of FP information exist in the settlements, the refugees reported that health workers were the main source of information for FP information [73.8%, *n* = 490]. A higher proportion of respondents reported that hospital is their main source of FP services [89.2%, *n* = 1169]. The majority who reported that hospital is the main source of FP services were from the host population [92.7%, *n* = 599] as compared to 85.8%, *n* = 570] of refugees.

### Current family planning use

Overall, the study revealed that 30.2% of the population under study were using FP. The study further established that a third [28.2%, *n* = 447] of the refugee population were using family planning methods (Table 2).

**Table 2:** Family planning use among refugee populations in Adjumani district

Variables	Modern FP users/Total, N	% modern FP use	Crude OR (95% CI)	Adjusted OR (95% CI)	*p*-value
**Sex**					
Male	65/217	30.0	1	1	
Female	126/447	28.2	0.92(0.27–3.07)	1.08(0.64–1.82)	0.763
**Age (years)**					
15–24	49/201	24.4	1	1	
25–34	91/259	35.1	1.68(1.15–2.44)	1.54(0.91–2.63)	0.111
35–45	34/130	26.2	1.09(0.52–2.29)	1.03(0.32–3.38)	0.955
45–54	12/56	21.4	0.85(0.12–5.81)	0.68(0.09–4.97)	0.707
55 +	5/18	27.8	1.19(0.06–22.11)	0.92(0.05–16.8)	0.957
**Marital status**					
Never married	26/119	21.8	1	1	
Married/Monogamy	141/411	34.3	1.87(1.26–2.76)	1.38(0.74–2.58)	0.312
Married/Polygamy	19/101	18.8	0.83(0.29–2.39)	0.67(0.32–1.42)	0.297
Widowed/Divorced/ Separated	5/33	15.2	0.64(0.34–1.19)	0.49(0.24–1)	**0.049**
**Highest education level**					
None/ Incomplete primary	57/257	22.2	1	1	
Completed Primary	49/137	35.8	1.95(0.88–4.33)	1.65(1.27–2.16)	** < 0.001**
Secondary +	85/270	31.5	1.61(0.98–2.66)	1.14(0.57–2.27)	0.715
**Religion**					
Catholic	56/249	22.5	1	1	
Protestant	78/226	34.5	1.82(0.80–4.12)	2.31(0.61–8.72)	0.216
Pentecostal	17/54	31.5	1.58(0.99–2.52)	1.56(0.61–3.94)	0.351
Moslem	13/38	34.2	1.79(0.32–9.99)	1.84(0.32–10.5)	0.492
SDA	4/14	28.6	1.38(0.62–3.07)	1.04(0.48–2.26)	0.927
ECS	23/83	27.7	1.32(0.75–2.32)	1.77(0.91–3.45)	0.094
**Occupation**					
Peasant	114/506	22.5	1	1	
Semiskilled	66/130	50.8	3.55(0.49–25.67)	3.55(0.75–16.82)	0.11
Skilled	11/28	39.3	2.22(1.70–2.91)	2.28(2.11–2.47)	< 0.001
**Duration in study area (years)**					
< 3	17/73	23.3	1	1	
3-5yrs	120/325	36.9	1.93(1.68–2.22)	2.24(1.46–3.42)	< 0.001
6 + yrs	54/265	20.4	0.84(0.29–2.38)	0.9(0.28–2.92)	0.862
**Desire a (another) child**					
No	25/127	19.7	1	1	
Yes	166/537	30.9	1.83(1.14–2.93)	1.14(0.64–2.02)	0.658

It was revealed that use of family planning amongst refugees was lower. However, the use of FP amongst the respondents in the age category of 25–34 years was at 35.1% as compared to those in the age category of 15–24 years that was at 24.4%. it was revealed that amongst the refugee populations, respondents who completed primary education were about twice more likely to use FP methods. The study further revealed that a significantly higher proportion of refugees who were skilled [39.3%, *n* = 28] were more likely to use family planning methods. This study shows that more than a third of the respondents [120/325] who have stayed in the area for 3–5 years were more likely to use FP methods. The study revealed that more than a third of refugees [32.6%, *n* = 159] would like to have another child. (Table 2).

This study established that a third [32.2%, *n* = 125] of the host population were using family planning methods (Table 3). The study further showed that FP use was more significant amongst respondents of ages above 55 years [61.5%, *n* = 26] as compared to the much younger age categories. We established that a significantly higher proportion of respondents [43.6%, *n* = 39] who are widowed/divorced/separated from the host population were using family planning methods. We established that a significant proportion of respondents from the host population [32.6%, *n* = 488] wanted to have another child.

**Table 3:** Family planning use among host populations in Adjumani district

Variables	Modern FP users/Total, N	% of modern FP use	Crude OR (95% CI)	Adjusted OR (95% CI)	*p*-value
**Sex**					
Male	67/258	26	1	1	
Female	125/388	32.2	1.35(0.3–6.14)	2.29 (0.61–8.52)	0.218
**Age (years)**					
15–24	45/159	28.3	1	1	
25–34	71/250	28.4	1.01(0.41–2.49)	0.89 (0.46–1.73)	0.732
35–45	45/154	29.2	1.05(0.65–1.67)	1.26 (0.98–1.60)	0.068
**45–54**	15/57	26.3	0.91(0.50–1.62)	1.77 (1.07–2.92)	**0.026**
**55 + **	16/26	61.5	4.05(0.63–26.03)	20.38(6.43–64.57)	< 0.001
**Marital status**					
Never married	10/42	23.8	1	1	
Married/Monogamy	139/470	29.6	1.34(1.08–1.67)	0.91(0.51–1.61)	0.742
Married/Polygamy	26/95	27.4	1.21(0.19–7.33)	0.61(0.17–2.26)	0.462
Widowed/Divorced/ Separated	17/39	43.6	2.47(1.29–4.73)	2.45(1.56–3.84)	** < 0.001**
**Highest education level**					
None/ Incomplete primary	79/290	27.2	1	1	
Completed Primary	37/162	22.8	0.79(0.42–1.50)	0.69(0.32–1.5)	0.35
Secondary +	76/194	39.2	1.72(0.71–4.18)	1.6(0.68–3.74)	0.277
**Religion**					
Catholic	127/472	26.9	1	1	
Protestant	41/109	37.6	1.64(0.65–4.14)	1.53(0.81–2.91)	0.189
Pentecostal	14/32	43.8	2.11(0.58–7.63)	2.15(0.55–8.41)	0.273
Moslem	5/13	38.5	1.7(0.18–16.14)	1.13(0.27–4.78)	0.864
SDA	5/20	25	0.91(0.23–3.59)	1.25(0.23–6.73)	0.792
**Occupation**					
Peasant	125/473	26.4	1	1	
Semiskilled	53/136	39	1.78(0.49–6.39)	1.66(0.53–5.24)	0.385
Skilled	14/37	37.8	1.69(0.57–5.03)	1.12(0.31–4.05)	0.865
**Duration in study area (years)**					
< 3	21/66	31.8	1	1	
3-5yrs	22/77	28.6	0.86(0.15–5.02)	0.93(0.16–5.49)	0.937
6 + yrs	145/489	29.7	0.90(0.22–3.66)	1.21(0.53–2.77)	0.644
**Desire a(another)child**					
No	33/158	20.9	1	1	
Yes	159/488	32.6	1.83(0.83–4.04)	3.73(1.45–9.60)	**0.006**

## Factors associated with family planning use among refugees and host populations

### Factors associated with family planning use among refugee population

In Table 2, the factors significantly associated with FP use amongst refugee populations included education, occupation, and duration of stay in the study area. Respondents who completed primary education were about two times more likely to use FP compared to those with no education; AOR = 1.65, 95% CI [1.27–2.16]. The use of FP was higher among those who had skills compared to those who were peasants, AOR = 2.28, 95% CI [2.11–2.47]. We further established that respondents who had stayed in the study area between 3–5 years were two times more likely to use FP methods; AOR = 2.24, 95% CI [1.46–3.42].

Main reasons for not using FP methods amongst refugee population was not wanting to use FP methods due to socio cultural norms and side effects of FP methods. In the Table 2 above, the raw percentage shows the proportion of users- presented from refuge populations. Therefore, study limitation is attributed to small sample size for some variables.

### Factors associated with FP use among host population.

In this study, we established that age, marital status, and desire for another child were significant factors associated with FP use amongst the host population. We found that respondents who were 45 years and above were about two times more likely to use FP methods; AOR = 1.77, 95% CI [1.07–2.92]. We also established that women who lived singly were over two times more likely to use family planning methods, AOR = 2.45, 95% CI [1.56- 3.84]. This study further established that respondents amongst the host population who had desire for another child were about four times more likely to use FP methods, AOR = 3.73, 95% CI [1.45- 9.60].

The key barrier associated with FP use amongst the host population was harassment of women and separation/divorce for not consulting the family members and side effects of FP methods.

In Table 3 above, the raw percentage included, shows the proportion of users presented from both populations. Therefore, study limitation is attributed to small sample size for some variables. We also established that another reason for not using FP methods among the host population were associated with age. The younger the respondent, the more unlikely to use FP method [28.4%, *n* = 71], compared to the older respondents of 55 years and above [61.5%, *n* = 16].

## Discussion

It is worth noting that family planning use depends on several factors. This study revealed current low contraceptive use amongst both refugee and host populations. The study further established that factors significantly associated with FP use among both populations included age, level of education, marital status, occupation, desire for another child and duration of stay in the study area. However, the commonest factors among both populations were marital status, thus women who were widowed/divorced/separated were more likely to use family planning methods.

### Current family planning use

This study found that about a third of refugees and the host population were using FP methods. We found that modern Contraceptive Prevalence Rate (mCPR) was 30.2% (32.2% host and 28.2% refugees). The mCPR for the refugees in Adjumani [28.2%] is higher than the mCPR back home in South Sudan [2.7%] with high unmet need of family planning [30.8%] and maternal mortality ratio of 789/100,000 live births [25]. The high mCPR amongst the refugees in Uganda could be attributed to the high exposure to FP information and services in the settlements provided by several partners through community outreaches [26].

For the host population, however, the mCPR is lower than the national mCPR of 38% [13]. Therefore, efforts to reduce the unmet need for FP in the west Nile region that has had persistent and highest unmet need for FP in the country (43%) compared to national average of 22% has been slow [17]. This may be attributed to poverty as women in the west Nile fall in the lowest wealth quintile. This finding concurs with a study by [27, 28] which reported that unmet need due to various reasons decreases with increasing wealth, from 37% among women in the lowest wealth quintile to 22% among women in the highest wealth quintile.

### Factors associated with family planning use

The factors significantly associated with FP use amongst refugee populations included age, marital status, education, occupation, desire to have another child and duration of stay in the study area. The study found that many of the refugees [71.8%, *n* = 321] are not currently using any FP method.

### Age

While age was a significant factor for family planning use, it was only among the older category of above 55 years who were able to decide to use FP. However, it is known that this age category is not very crucial for FP use due to the fact about reaching menopausal period. The younger the respondent, the more unlikely to use FP method [28.4%, *n* = 71] compared to the older respondents of 55 years and above [61.5%, *n* = 16]. In another study, the finding is not in line with our study finding because in Ethiopia, it is the young women of 18–20 years who have more decision making power for FP use [29].

### Marital status

The study also revealed that other factors significantly associated with family planning use included marital status. Although some of the barriers associated with FP use amongst the host population was harassment of women and separation/divorce for not consulting the family members, this study established that women who lived singly were over two times more likely to make decision to use family planning methods, compared to those in union/married. This could be due to the autonomy that women have and are empowered to take control of their sexual life and agency. This study was in congruent with a study done in Ghana, Argentina and India [30] which showed that women who are more likely to use FP have a higher decision making autonomy.

### Education

This study revealed that respondents who completed primary education were 1.65 times more likely to use FP compared to those with no education. We established that the low educational level has exacerbated the fear to use contraception given the notion that the elders believe in their cultural norms that women are to produce more children in compensation for the dowry paid to marry them. Due to low educational level, the women are not empowered enough to make their own decision rather rely on the men to make final decision on their health issues. However, the refugees who atleast completed primary level education were more likely to use FP. This concurs with several studies [31–33] which showed that education has the greatest influence on FP use because of knowledge, empowerment, economic status and autonomy hence increased ability to maintain FP method. This demonstrates the fact that low educational level or no education is associated with less empowerment to make own decision but rather rely on a husband or believing on cultural norms. Thus, education is crucial to accelerate women empowerment for their health needs. This finding concurs with a study done by [34–36] who reported that socio cultural factors affect use of FP despite strong interest of individuals to use it because of low educational level and over believing in cultural norms.

### Occupation

We also established that women who acquired some skills were 2.28 times more likely to use FP. This is attributed to the ability to provide for their basic needs should any conflict arise. Therefore, the decision to use FP was higher among those who were engaged in some income generation due to their skills compared to those who were peasants. This finding concurs with a study conducted in Burkina Faso which revealed that women who have some form of income generation are empowered to make decision to use modern FP methods [32]. Therefore, it is crucial to design interventions that are self-centered around women and girls to increase their self-efficacy and agency.

### Duration of stay in the study area

Our study established that respondents who had stayed in the study area between 3–5 years were over two times more likely to use FP methods. While on transition, refugees need more protection due to sexual violence that happens during the movement in search for safety due to conflict in home country. It is evident that women have no access to FP services because they are not only unfamiliar with the environment but also on mobility. This finding is supported by studies done in Turkiye and Lebanon [37, 38] which revealed that refugee women during mobility have little or no access to FP services. Some of the reasons are language barrier, limited information about FP, no method of choice among others. This could be due to other social services like food, shelter and security that are prioritizes [7, 39]. Secondly, it could be due to limited quality health services, myths and misconceptions as factors that affected FP use at the time of relocation to another country before being settled in a host community. This concurs with several studies [39–41] which revealed that weak health system and poor health services affect the use of FP.

### Desire to have another child

In this study, it was statistically significant that majority of the women who were using FP had the desire for another child. This implies that their decision to use FP methods was for spacing purpose. This could be due to previous experience of too soon child births and thus the desire to space. Our finding is in congruent with a study done in Nigeria [42] which revealed that child spacing is the most important benefit of FP.

### Other common factors associated with FP use among both populations

Our study revealed that husband disapproval was one of the factors affecting FP use. Infact, women who made own decision to use FP methods and did not consult key family members including their husbands prior to the use of FP, were harassed, and chased away from their marital home (separated/divorced). This has caused fears in many women and thus refrained from using FP methods. In a study done [43] among the Fertit ethnic group in South Sudan, revealed that men are sole decision-makers at households and this is not questionable. This is in agreement with other studies done in Africa by several scholars [44–46] who reported unequal gender relations and partner disapproval affecting use of FP. Due to that fear, majority of the women were not using FP services.

## Conclusion and recommendations

The study has revealed low family planning use among both refugees and host population in Adjumani district. The main factors associated with decision to use FP amongst both populations included age, level of education, marital status, occupation, desire to have another child and duration of stay in the study area. Over 70% of the women among both populations have never used any FP methods. Main reasons for not using FP methods included fear of harassment by the family members. Therefore, there is need to sensitize both communities about the benefits of FP and promote couple counseling at community level. Thus, the need for conducting outreaches to create more awareness on the benefits of FP as well as offer FP services to women of reproductive age.

## Data Availability

The datasets used were analyzed during the current study and are available from the corresponding author on reasonable request.
